# Lysosomal dysfunction in diabetes and diabetic complications

**DOI:** 10.3389/fendo.2026.1794600

**Published:** 2026-04-07

**Authors:** Xin Chen, Lishan Huang, Zhiwen Xiao, Libin Liu

**Affiliations:** Department of Endocrinology, Fujian Medical University Union Hospital, Fuzhou, China

**Keywords:** autophagy, diabetes, diabetic complications, lysosomal, lysosomal dysfunction

## Abstract

Lysosomes, as organelles with degradative, secretory and signaling functions in eukaryotic cells, play a pivotal role in maintaining cellular energy homeostasis and biological recycling processes. In recent years, lysosomal dysfunction has garnered extensive attention from scholars for its implications in neurodegenerative and autoimmune diseases. however, its role in the occurrence and progression of diabetes mellitus and its complications remains to be further explored. Therefore, this article summarizes the research progress on lysosomal dysfunction in diabetes and its complications, hoping to highlight a promising therapeutic direction.

## 1 Introduction

Globally, diabetes is expected to affect 783 million people in 2045 (1). Thus, preventing diabetes mellitus (DM) and its associated complications has become a primary goal of public health authorities globally. A growing area of research focuses on understanding the cellular mechanisms involved in DM, particularly how cellular organelles contribute to its progression and associated complications (2–4).One pivotal role in cellular dysfunction is the lysosome, an organelle that plays a vital role in maintaining cellular homeostasis through processes like cellular debris degradation and nutrient recycling (5).In addition, lysosomes are involved in processes such as membrane repair, immune signaling, bone resorption, and lysosomal exocytosis (6–8). Changes in lysosomal function are considered to be closely related to the pathogenesis of metabolic disorders and other diseases (9–11). In this review, we summarize the recent findings and discuss the role of lysosomal structural and functional changes in the pathogenesis of DM and its associated complications.

## 2 Overview of lysosomes

Structurally, lysosomes consist of a monolayer membrane containing hundreds of membrane proteins and an acidic lumen filled with soluble hydrolases. The acidic environment within lysosomes is typically in the pH range of 4.5-5.0 to maintain lysosomal hydrolase activity and autophagy (12). Among lysosomal transport proteins, V-ATPase maintains the activity of most lysosomal hydrolases by transporting hydrogen ions to acidify the lysosomal lumen. Moreover, the pH gradient generated by V-ATPase across different organelles is crucial for the maturation of endosomes from early to late stages and the subsequent fusion of late endosomes with lysosomes (13). Soluble hydrolases including proteases and lipases degrade various biomolecules and damaged organelles into lysosomes (14). Cathepsin D (CTSD), cathepsin B (CTSB), and cathepsin L (CTSL) are the most abundant and functionally critical lysosomal hydrolases (14). Their dysregulation have been widely implicated in the pathogenesis of neurodegenerative, renal, and cardiovascular diseases (15). All three primarily mediate intracellular protein degradation and turnover, thereby acting as central regulators of proteostasis and the normal physiological function of lysosomes (16). In addition, CTSD modulates the bioactivity of various enzymes and growth factors (17); CTSB mediates the release of inflammatory mediators and regulates programmed cell death (18); and CTSL plays a crucial role in immune regulation and programmed cell death (19). Oxidative stress and other stimuli damage lysosomal membranes, leading to the release of lysosomal contents including cathepsins such as CTSB into the cytosol. This process is termed lysosomal membrane permeabilization (LMP) (20). Massive lysosomal leakage induces cytosolic acidification, degradation of cellular components and necrosis, ultimately resulting in cell death (20).

Lysosome associated membrane proteins (LAMPs) include various structural proteins and transport proteins. Among the structural proteins, LAMP-1 and LAMP-2 of the LAMPs family are the main components of the lysosomal membrane, protecting the membrane from degradation by highly glycosylated structures (21). Other transport proteins, such as the Niemann-Pick type C1 protein (NPC1) facilitates the export of cholesterol from the lysosomes (22). Additionally, the lysosomal membrane expresses the two-pore channel (TPC), which regulates lysosomal Ca^2+^and Na^+^ transport by sensing nicotinic acid adenine dinucleotide phosphate (NAADP) signals (23). In particular, the lysosome is a Ca^2+^signalling hub, and lysosomal calcium signaling is critical for the regulation of core processes including lysosomal exocytosis, membrane fusion, and intracellular trafficking (24). Lysosomal calcium is primarily derived from calcium transport mediated by endoplasmic reticulum (ER)-lysosome membrane contact sites (25). Lysosomes also transmit signals to the ER via local Ca^2+^ release, triggering calcium-induced calcium release and amplifying the signaling cascade (26). The lysosome further interacts with mitochondria to regulate inter-organelle calcium flux (27); the lysosome, ER, and mitochondria form a synergistic regulatory network through inter-organelle membrane contact sites, to jointly maintain intracellular calcium homeostasis (24). In addition, lysosomal calcium signaling mediates the dynamic interaction between lysosomes and lipid droplets, regulates the lipophagy process, and synergistically maintains the overall metabolic homeostasis of cells (28). The key metabolic regulator mammalian target of rapamycin complex 1 (mTORC1) is activated on the lysosomal membrane (29). Decreased glucose levels inhibit mTORC1 via two pathways (30). One pathway relies on AMP-activated protein kinase (AMPK) phosphorylates tuberous sclerosis complex 2 to suppress mTORC1 activity, thereby promoting autophagy to restore cellular energy homeostasis. Besides, axis inhibition protein (Axin) translocates to lysosomes and binds to the Ragulator complex, ultimately resulting in the dissociation of mTORC1 from the lysosomal membrane and its subsequent inactivation. Notably, metformin and starvation act through the same lysosomal route by promoting the assembly of the Axin1/AMPK complex on the lysosomal membrane, thereby suppressing the activity of mTORC1 (31).

**Figure 1:**
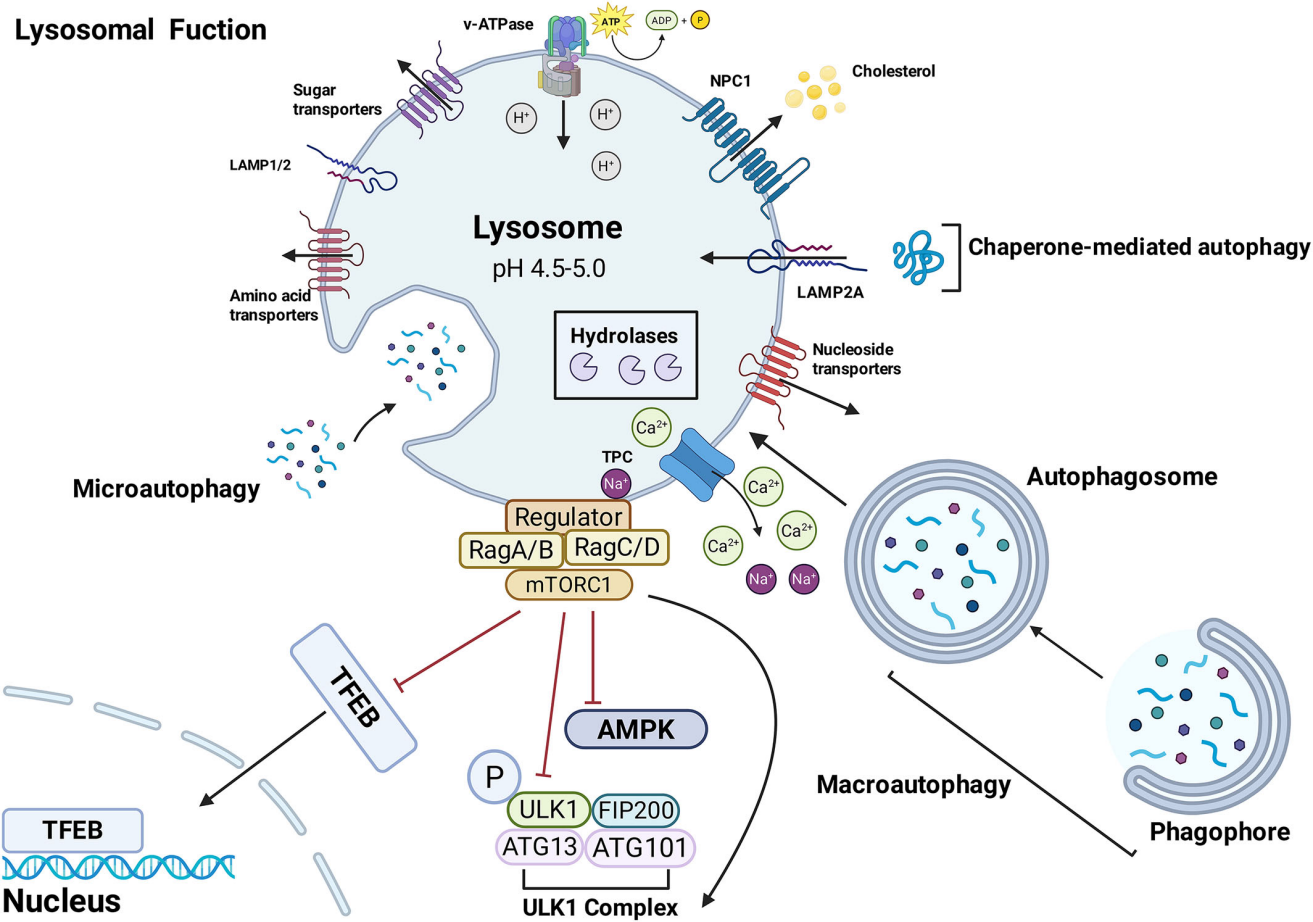
The Lysosome as a Signaling Hub in Autophagic Regulation and Cellular Homeostasis.

Functionally, lysosomes serve as signaling organelles that sense nutrient availability and participate in the degradation and recycling of intracellular and extracellular substances through endocytosis and autophagy to meet the cellular nutritional demands. Lysosomes also activate signaling pathways from lysosomes to the nucleus to regulate energy metabolism (32).Under nutrient-rich conditions, activated mTORC1 phosphorylates transcription factor EB (TFEB) and transcription factor binding to transcription factor E3 (TFE3), and sequesters them in the cytoplasm (33). Under energy stress, mTORC1 inactivation results in the dephosphorylation of TFEB and TFE3 and their translocation to the nucleus, thereby upregulating lysosomal biogenesis and autophagy (33). Lysosomal membrane proteins, soluble hydrolases, and signaling molecules are crucial to the normal function of lysosomes. Taken together, these interconnected functional attributes underscore the pivotal role of lysosomes in cellular physiology, and **Figure 1** summarizes the normal lysosomal function.

## 3 Lysosomal function and autophagy

Autophagy refers to the process of transporting cytoplasmic components to lysosomes for degradation (34). As a lysosome-dependent catabolic pathway, it enables cells to adapt to environmental changes, maintain cellular homeostasis and preserve cellular physiological functions. Autophagy is classified into three major types: macroautophagy (MA), microautophagy, and chaperone-mediated autophagy (CMA) (35). MA is the main form of autophagy, which responds to cellular stress induced by environmental changes and physiological signals. It forms autophagosomes with double membranes to encapsulate substances like damaged organelles and pathogens; these autophagosomes subsequently fuse with lysosomes to form autolysosomes and utilize lysosomal acid hydrolases for degradation. Autophagy functions as either a non-selective degradation process or a highly selective pathway (36). The process by which autophagosomes target and degrade specific cargos is termed selective autophagy (37). Depending on the substrate type, selective autophagy can be categorized into mitophagy, ER-phagy, lipophagy, and other forms (37). Consequently, lysosomal dysfunction leads to the accumulation of dysfunctional organelles, thereby disrupting intracellular homeostasis (38). Microautophagy refers to the process of direct engulfment and degradation of contents by lysosomes. CMA involves the transport of cytoplasmic substrates to the lysosomal lumen for degradation after binding with molecular chaperones (39). In summary, lysosomes are the essential organelles where different types of autophagy occur, and autophagy is closely related to lysosomal function. Meanwhile, as one of the primary functions of lysosomes, impaired autophagy can also induce lysosomal dysfunction.

Among lysosomal membrane proteins, LAMP2A, a subtype of LAMP-2, is a key CMA protein that transport cytoplasmic proteins with specific CMA motifs to lysosomes for degradation (40). LAMP2B, primarily expressed in cardiomyocytes, mediates autophagosome-lysosome fusion and thus participates in the MA, playing a critical role in mitochondrial and cardiac function regulation (40). Among lysosomal transport proteins, V-ATPase maintains the activity of most lysosomal hydrolases by transporting hydrogen ions to acidify the lysosomal lumen. NPC1 is a key cholesterol transporter on the lysosomal membrane. Mutations in the *NPC1* gene impair fusion between autophagosomes and lysosomes, leading to abnormal accumulation of cholesterol and lipids within lysosomes and triggering cell apoptosis. Clinically, this manifests as Niemann-Pick type C1 (NPC1) disease, a rare lysosomal storage disorder (41). The signaling molecule mTORC1 senses and responds to fluctuations in intracellular and extracellular nutrient availability to control cell growth; it also plays a vital role in regulating autophagy (42). Under nutrient-sufficient conditions, mTORC1 kinase inhibits autophagosome formation by phosphorylating sites on UNC-51-like kinase (ULK1) and autophagy-related gene 13 (ATG13). During amino acid deprivation, inactivation of mTORC1 anchored by lysosomes triggers the dephosphorylation of the ULK1 complex, which then recruits the phosphoinositide 3-kinase (PI3K) complex to promote autophagosome formation. Additionally, lysosomes can recruit adenosine 5’-monophosphate-activated protein kinase (AMPK) to mediate ULK1 phosphorylation, forming a synergistic regulatory network with mTORC1 signaling pathway. This coordinated regulation ensures rapid activation of the lysosomal-autophagic degradation pathway in cells during nutrient deprivation to maintain energy homeostasis (43). TPCs are believed to play a bidirectional regulatory role in autophagy in different cell models (44). In cancer cells, overexpression of TPC2 disrupts lysosomal acidification, impeding the fusion of autophagosomes with lysosomes and inhibiting autophagy (45); in contrast, the increased expression of TPC1/2 under starvation conditions positively regulate autophagy (46). *TPC* gene silencing impairs autophagic flux in cardiomyocytes. In TPC1/2 double knockout mice, the number of small, immature lysosomes in the heart is greater than that in wild-type mice. This ultimately results in decreased cardiomyocyte viability (46). In summary, lysosomal membrane proteins, hydrolases, signaling molecules, and lysosomal ion channels collectively regulate autophagy including autophagy initiation, autophagosome maturation, autophagosome-lysosome fusion, and degradation, thereby jointly regulating cellular metabolism homeostasis.

## 4 Lysosomal function and diabetes mellitus

DM is a metabolic disorder caused by impaired insulin secretion and/or action, frequently accompanied by dysregulated glucose and lipid metabolism. Lysosomes and autophagy interact to jointly maintain cellular nutritional balance. Autophagy is considered to preserve pancreatic β-cell function, while lysosomal dysfunction in β-cells is considered a key pathogenic mechanism of type 2 diabetes mellitus (T2DM) (47). How lysosomal dysfunction mediates the development of T2DM by impairing β-cell function thus warrants further investigation. Studies have indicated that long-term exposure of β-cells to metabolic stress environments, specifically glycotoxicity/lipotoxicity, elevates lysosomal pH, leading to impaired autophagosome-lysosome fusion in β-cells (48). This suggests that lysosomal dysfunction may induce β-cells apoptosis by disrupting autophagy. Additionally, elevated lysosomal pH impairs autophagy, leading to the accumulation of damaged mitochondria and reduced cellular ATP production. This results in the accumulation of insulin secretory vesicles within β-cells and impaired glucose-stimulated insulin secretion. These processes collectively contribute to glucose dysregulation (49). Further studies have revealed that impaired autophagy flux induced by lysosomal dysfunction in β-cells of NPC1 knockout mice leads to impaired β-cells differentiation, proliferation, and mitochondrial oxidative phosphorylation capacity (50). Meanwhile, Mendelian randomization studies have revealed that the higher NPC1 expression levels were strongly correlated with a low incidence of diabetes in pregnancy, implicating NPC1 as a genetic predictor for gestational diabetes onset (51). Notably, the lysosome-resident ion channel TPC plays a vital role in β-cells Axin secretion by regulating Ca^2+^ transport (52). Knocking out the *TPC1/2* gene in mouse models resulted in impaired glucose-stimulated insulin secretion in β-cells (53, 54). In research on the Chinese population, it has been found that variations in the *TPCN2* gene encoding TPC2 are associated with T2DM and impaired β-cell function (55). However, genome-wide association study (GWAS) data from populations of European descent have shown that *TPCN2* genetic variants exhibit only weak associations with fasting insulin and homeostatic model assessment of insulin resistance (HOMA-IR), without reaching genome-wide significance (54). Therefore, these variants are not yet considered as robust metabolic genetic loci. Collective evidence from existing genetic and functional studies suggests that TPC dysfunction may contribute to the pathogenesis and progression of diabetes, warranting further in-depth and systematic investigations in diverse populations. β-cells possess a direct lysosomal degradation pathway for nascent insulin granules which is known as stress-induced nascent granule degradation (SINGD) (56). This process occurs independently of autophagosome formation. Under physiological conditions, the protein kinase D-mTORC1 axis precisely regulates SINGD, maintaining insulin secretion homeostasis while negatively regulating autophagy (57). This constitutes a core mechanism for sustaining β-cells homeostasis. Metabolic stress like DM leads to excessive activation of the SINGD pathway, causing massive loss of insulin granules (57). Meanwhile, SINGD inhibits autophagy by activating mTORC1, exacerbating β-cells failure and accelerates the progression of T2DM. SINGD represents a key pathway through which lysosomal dysfunction mediates β-cells injury in diabetes.

In addition, lysosomal dysfunction may contribute to the DM pathogenesis by disrupting cellular energy metabolism. LAMP-2 is a key lysosomal membrane protein, mediates the fusion of autophagosomes-lysosomes and subsequent degradation. Yamahara et al. found that *LAMP-2* gene deficient mice prevented high-fat diet induced T2DM by increasing energy expenditure, suggesting that LAMP-2 may be a key protein in revealing the pathogenesis of T2DM induced by obesity (58). Studies in aging mice revealed that calorie restriction activates CMA by stabilizing the lysosomal LAMP-2 membrane protein, which may explain how calorie restriction protects against the progression of diabetes (59). The bidirectional regulation of cellular energy by LAMP-2 in pathological and physiological conditions indicates that it is a critical lysosomal target for intervening in T2DM development. Among lysosomal transport proteins, V-ATPase regulates glycolysis, insulin secretion, and glucose transporters through its lysosomal acidification function (60). In human cells, V-ATPase inhibition upregulates glycolysis by activating hypoxia-inducible factor 1α (HIF-1α) (61). In T2DM rat models, increased expression of V-ATPase in pancreatic islet cells was associated with a reduction in insulin granules; meanwhile, the V-ATPase inhibitor bafilomycin B1 improved blood glucose levels under starvation by reducing V-ATPase activity (62). Additionally, metformin, a widely used antidiabetic drug, has been shown to inhibit V-ATPase activity by binding to the presenilin enhancer 2 protein on the lysosomal membrane. This complex recruits the V-ATPase subunit ATP6AP1, thereby triggering the translocation of AXIN and liver kinase B1 to the lysosomal membrane. This activates the AMPK signaling pathway, ultimately reducing postprandial blood glucose and hepatic lipid deposition (63). Thus, V-ATPase modulates insulin secretion and the AMPK signaling pathway by regulating lysosomal acidification, and its dysfunction promotes the pathogenesis and progression of T2DM (64). The mTORC1 signaling complex, a key regulator of cellular nutrient metabolism, exhibits altered assembly, activity, and lysosomal localization in response to changes in glucose levels. Under glucose deprivation, mTORC1 can be inhibited in an AMPK-dependent or independent manner. Under hyperglycemia conditions, pancreatic islet β-cells exhibit increased mTORC1 activity and decreased mTORC2 activity. Inhibition of the mTORC1-Ribosomal protein S6 kinase beta-1 signaling pathway can restore insulin secretion, indicating that elevated mTORC1 impairs β-cell function (65). In addition, mTORC1 downstream effectors are also involved in the regulation of adipogenesis (66). Thus, dysregulation of the mTOR signaling leads to glucose and lipid metabolism dysfunction and the development of insulin resistance (67). In summary, lysosomal dysfunction promotes the pathogenesis and progression of DM by impairing pancreatic β-cell function and cellular energy metabolism.

Meanwhile, given the complexity of lysosomal structure, especially lysosomal proteases which include CTSL, CTSB and CTSD that are the most abundant and functionally potent lysosomal hydrolases, although numerous studies have linked elevated levels or enhanced activities of these cathepsins to hyperglycemic and diabetic conditions, direct evidence linking these proteases to diabetic pathogenesis remains elusive (68, 69). However, in mice with pancreas specific knockout of CTSL, CTSB, and CTSD, CTSB/CTSD double-knockout mice exhibited impaired autophagy in pancreatic acinar cells, thereby inducing chronic pancreatitis (70). Therefore, the roles of CTSB and CTSD in DM pathogenesis warrant further investigation.

## 5 Lysosomal function and chronic complications of diabetes

**Figure 2:**
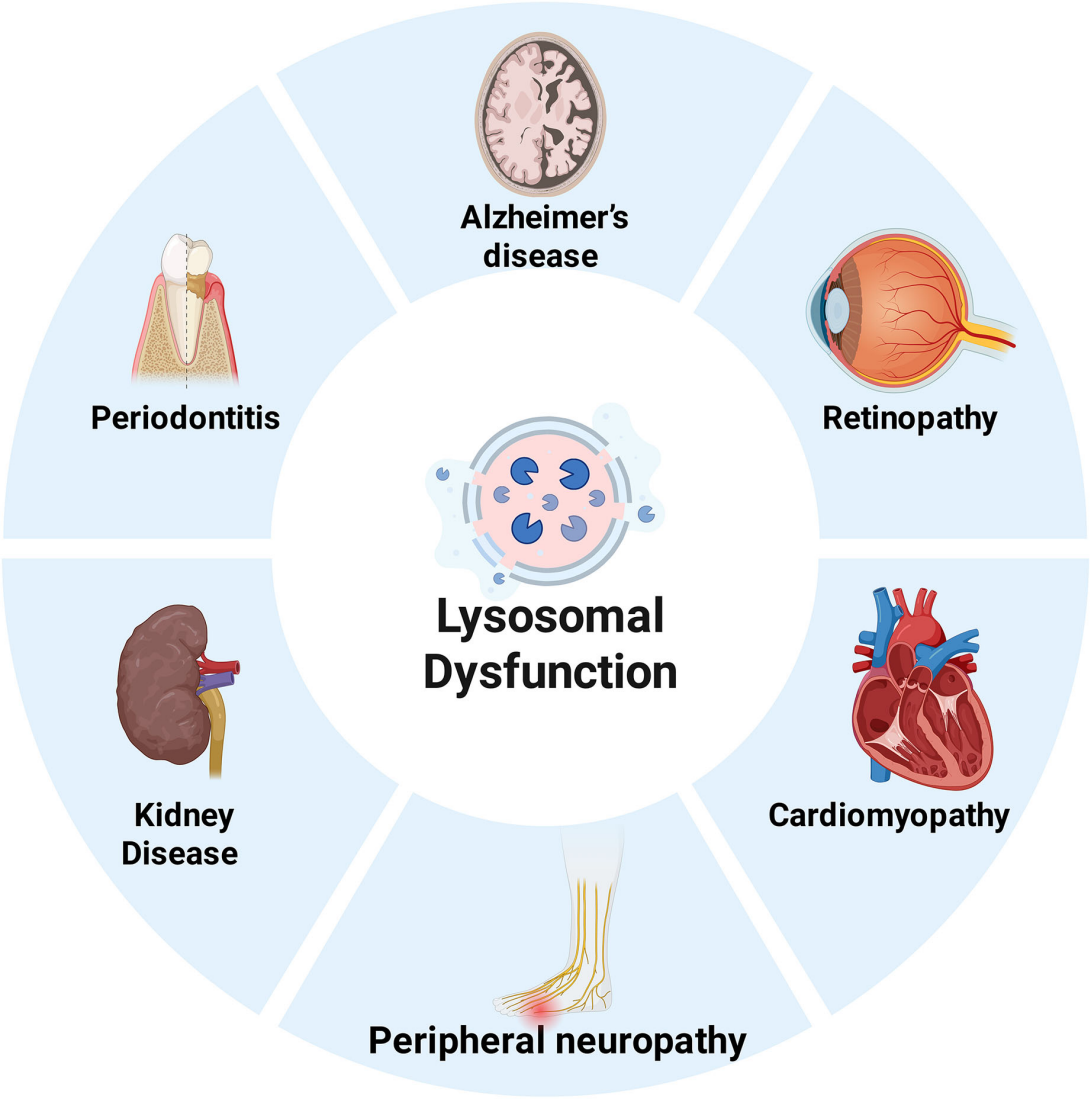
Lysosomal Dysfunction in Diabetic Complications.

Brownlee’s hypothesis postulates that hyperglycemia leads to elevated levels of reactive oxygen species through multiple pathways, including the polyol pathway, advanced glycation end products (AGEs) pathway and other mechanisms, which is a key underlying mechanism of chronic diabetic complications (71). However, clinical studies have shown that strict glycemic control fails to prevent or reverse the progression of these complications. Given that Brownlee’s hypothesis failed to fully explain the pathogenesis of chronic complications in diabetes, there is an urgent need to identify novel molecular targets involved in their pathological and progression. Our previous studies have demonstrated that mitophagy plays a critical role in diabetic myocardial injury and diabetic cognitive dysfunction (72, 73). The key steps of autophagy involve autophagosome-lysosome fusion and subsequent degradation. Therefore, exploring the association between lysosomal function and chronic diabetic complications holds important reference value. **Figure 2** summarizes the key impact of lysosomal dysfunction on diabetes and its associated chronic complications.

### 5.1 Diabetic cardiomyopathy

Diabetic cardiomyopathy (DCM) refers to myocardial systolic and/or diastolic dysfunction in diabetes (74). DCM is a major cause of heart failure and mortality in patients with DM. Current studies have shown that the pathogenesis of DCM involves dysfunctional autophagosomes and mitochondria, which subsequently induce myocardial vascular damage and ultimately impair cardiac function (75). However, autophagic alterations in DCM may depend on alterations in lysosomal activity. Therefore, changes in lysosomal function in DCM also deserve further investigation. The lysosomal proteases CTSL and CTSB, which are involved in the degradation of intracellular and extracellular proteins and the release of amino acids for cellular utilization, are considered as potential key contributors to several cardiovascular diseases (76). Elevated CTSB protein levels have been observed in a DCM mouse model (77). Additionally, CTSB induces myocardial injury by activating NOD-like receptor protein3 (NLRP3) mediated cardiomyocyte pyroptosis and downstream inflammatory cascades, thereby exacerbating structural and functional dysfunction of DCM (77). Furthermore, previous studies in macrophages have demonstrated that elevated CTSB acts as an upstream signal to regulate the expression of lysosomal and autophagy-related proteins, negatively regulating the biogenesis and size of lysosomes and autophagosomes and thus impairing autophagy (78). Whether CTSB regulates lysosomal and autophagosomal homeostasis in DCM through the TFEB and mTORC1 signaling pathways warrants further investigation. In addition, elevated expression of CTSD and pro-apoptotic protein Bid has been observed in cardiomyocytes of DM mice, leading to cardiomyocyte death. This may be associated with the abnormal distribution of CTSD caused by LMP (79).

Lysosomal neuraminidases 1 (NEU1), which mediates the degradation of sialic acid residues and regulates glucose metabolism, is upregulated in cardiomyocytes of diabetic rats. Inhibition of NEU1 alleviates myocardial fibrosis, inflammatory, apoptosis, and oxidative stress in diabetic mice through the sirtuin 3 (SIRT3) signaling pathway (80). LAMP-2, a pivotal lysosomal membrane protein that maintains lysosomal membrane stability and mediates autophagy, exhibits decreased expression in the myocardium of T2DM mice, accompanied by increased cardiomyocyte apoptosis. LAMP-2 overexpression can reverse diabetic cardiac injury (81). These lysosomal-related pathways can independently induce myocardial injury, while saturated fatty acids may synergistically exacerbate myocardial injury by downregulating the expression of TFEB, a major regulator of lysosomal biogenesis, thereby reducing CTSB activity and LAMP2A expression (82).

In conclusion, lysosomal dysfunction is a key pathological feature of DCM, manifested by abnormal expression of hydrolases (CTSB, CTSD, NEU1) and membrane proteins (LAMP-2), which synergistically damage the myocardium through multiple pathways including impairing autophagy, inducing inflammation, promoting pyroptosis, and exacerbating oxidative stress. Therefore, enhancing lysosomal quality control is an important strategy to prevent and intervene in the pathogenesis and progression of DCM. Notably, various fasting regimens have emerged as a promising strategy to prevent and even reverse cardiometabolic diseases (83). Intermittent fasting maintains cellular and systemic homeostasis during the feeding-fasting cycle by activating autophagy and regulating the TFEB−mTORC1 pathway, ultimately mitigating pathological damage associated with cardiometabolic disorders (84).

### 5.2 Diabetic kidney disease

Diabetic kidney disease (DKD), the most common microvascular complication of diabetes, is a major cause of end-stage renal disease (85). Compared with other organs, the kidneys is enriched with lysosomes. Exposure to hyperlipidemic and/or hyperglycemia conditions results in the downregulation of lysosomal hydrolytic enzyme expression and impaired enzymatic maturation, thereby compromising lysosomal degradative function, exacerbating renal cell injury, and ultimately inducing renal cell apoptosis. Therefore, the pathogenic role of lysosomal dysfunction in DKD warrants further exploration (86, 87).

Podocytes, the visceral epithelial cells of the glomerular basement membrane, are crucial for maintaining glomerular filtration function. The cell bodies of podocytes are abundant in lysosomes, whereas only a small number of lysosomes are present in their foot processes (88). Under normal physiological conditions, podocytes degrade endocytosed albumin through lysosomes to prevent the clogging of the glomerular filtration barrier (89). In the course of DKD progression, pathological changes in podocytes from hypertrophy to progressive loss can be observed (90). Loss of podocyte foot processes is closely associated with the development of proteinuria and nephrotic syndrome. Podocyte foot process effacement and collapse of the slit diaphragm formed by podocyte foot processes attached to the glomerular basement membrane, are closely associated with the development and progression of proteinuria and nephrotic syndrome (91). Late-stage DKD is typically characterized by massive proteinuria, indicating the critical role of podocytes in DKD pathogenesis (92). As essential components of the glomerulus, mesangial cells (MC) not only regulate capillary blood flow and ultrafiltration surface area, but also maintain extracellular matrix homeostasis via their abundant lysosomal hydrolases (88). Restoration of impaired mitophagy mediated by PINK1-Parkin in MCs has been demonstrated to attenuate renal injury in DKD (93). Nevertheless, direct studies focusing on lysosomal dysfunction in MCs in DKD remain limited. Given the close association between lysosomal function and mitophagy, the role of MC lysosomal function in DKD pathogenesis warrants further investigation.

Lysosomal acid ceramidase (AC), which mediates the degradation of ceramide, can alleviate lipid toxicity in DKD and is regarded as essential for maintaining podocyte structural and functional integrity (94). In DM mice, AC activity is decreased, and upregulation of AC activity alleviates glomerular endothelial cell and podocyte injury (95). Additionally, podocyte-specific CTSD-knockout mice exhibited glomerulosclerosis and proteinuria, suggesting that normal CTSD function in podocytes is essential for maintaining the structure and function of the glomerular filtration barrier (96). Liu et al. further found that AGEs can induce podocytes oxidative stress, leading to LMP and CTSD release, which subsequently impairs podocyte autophagy and ultimately renal cell injury (97). This indicates that abnormal release and localization of CTSD can also contribute to DKD pathogenesis. Another lysosomal hydrolase, CTSL, mediates the degradation of proteins essential for maintaining podocyte foot process morphology. Studies have found that CTSL expression is upregulated in podocytes of patients with DKD (98). CTSL-deficient mice can maintain normal renal function. Indicating that CTSL plays a crucial role in the pathogenesis of experimental DKD (99). These results suggest that both hyperglycemia and hyperlipidemia in DM can impair the function of lysosomal hydrolases in podocytes, thereby inducing podocyte injury. Among lysosomal transport proteins, V-ATPase plays a major role in maintaining the acidic environment of lysosomes. Studies have shown that the acidic environment of lysosomes can promote exosomes secretion, and abnormal exosome secretion may induce podocyte dysfunction in DKD. Loss of SIRT1 inhibits lysosomal acidification by downregulating the expression of the V-ATPase A subunit in renal podocytes. SIRT1 overexpression can significantly restore lysosomal acidification, inhibit exosome secretion, and alleviate podocyte injury (100). Notably, AC modulates the interaction between podocyte lysosomes and multivesicular bodies, as well as release of exosomes, by mediating transient receptor potential channel-dependent Ca_2+_ mobilization (101). Further investigations are needed to elucidate whether reduced AC activity in DM mice induces podocyte dysfunction by dysregulating exosome secretion.

In addition, accumulating evidence indicates that renal tubules, while being affected by glomerular injury, are also involved in the progression of DKD. Under physiological conditions, proximal tubules, particularly the S1 segment, are abundant in lysosomes (102). Cathepsins L, B, and D within lysosomes mediate the degradation of endocytosed proteins during autophagy (102). Lysosomal dysfunction can further induce renal tubular inflammation and fibrosis (103). Elevated urinary excretion of cathepsins caused by proximal tubule injury also serves as an important biomarker for renal damage (104). In DKD, Mothers Against Decapentaplegic Homolog 3 (SMAD3) protein inhibits TFEB expression, thereby suppressing lysosome biogenesis and causing lysosome depletion in renal tubular epithelial cells. This leads to autophagy dysregulation and promotes the progression of DKD (105). In summary, lysosomes represent a pivotal target for mediating podocyte and renal tubular epithelial cell injury in DKD. Dysregulated lysosome function can directly or indirectly induce podocyte and renal tubular epithelial cell injury by impairing autophagy. Therefore, clearing dysfunction lysosomes or restoring the normal physiological functions of lysosomes may serve as a potential and potent approach for the prevention and treatment of DKD (106).

### 5.3 Diabetic peripheral neuropathy

Diabetic peripheral neuropathy (DPN) is one of the most common chronic complications of diabetes. Hyperglycemia is a major risk factor for DPN, and AGEs play a pivotal role in the occurrence and development of diabetic chronic complications. Zhou et al. found that the natural small molecule schisandrin A can activate V-ATPase-dependent lysosomal acidification by targeting the cysteine 335 residue of ATP6V0D1, thereby mediating the allosteric change of the V-ATPase subunit ATP6V0D1 and protecting against AGEs-induced neuronal apoptosis (107). Lysosomes are dynamic organelles, in which both hyperactivated and impaired lysosomal function can contribute to the pathogenesis of various diseases. Upregulation of the hydrolase CTSB can enhance inflammatory response and induce neuronal death, and increased CTSB expression at both the transcriptional and translational levels can be observed in several neurodegenerative diseases (108). Under a high-fat diet, compared with wild-type mice, mice with Schwann cell−specific p75 neurotrophin receptor knockout exhibited significantly elevated mRNA and protein levels of CTSB in C-fiber axoplasm, together with markedly increased abundance of lysosomes and phagosomes (109). These findings implicate that the lysosomal dysfunction may be involved in the pathogenesis of DPN. Meanwhile, in the diabetic state, loss of p75 neurotrophin receptor in Schwann cells induces axonal atrophy and C-fiber loss by upregulating CTSB and exacerbating lysosomal stress. C-fibers are small unmyelinated fibers responsible for the conduction of pain and temperature sensation. Their loss in diabetes patients leads to paresthesia and allodynia, which significantly increases the risk of diabetic foot ulcers and lower-limb amputations, ultimately impairing patients’ quality of life (110). In summary, both hyperglycemia and hyperlipidemia in DM can trigger lysosomal dysfunction and induce the occurrence of DPN. Current mechanistic research on the relationship between DPN and lysosomal dysfunction remains limited, and further research is warranted to elucidate their underlying functional crosstalk.

### 5.4 Cognitive dysfunction in diabetes

DM is an independent risk factor for cognitive dysfunction. Alzheimer’s disease (AD), the major subtype of dementia, is the most serious stage of diabetes related cognitive dysfunction (111). Studies have shown that the risk of developing AD in patients with DM is 1.5 -2.5 times higher than in patients without DM (112). Notably, AD has recently been defined as the “type 3 diabetes”, further underscoring the intimate connection between diabetes and AD pathogenesis (113). Pathologically, the main changes in AD include the deposition of amyloid beta (Aβ) and the excessive phosphorylation of microtubule associated protein Tau (Tau), which cause neuronal toxicity through the generation of oxygen free radicals and other pathways. Morphologically, immature autophagic aggregates can be observed in the brains of patients with AD (114). Based on the fact that lysosomes are the main site for Aβ degradation and their importance in the autophagy, the role of lysosomes in AD among patients with DM deserves further exploration.

T2DM induces insulin resistance in various tissues, of which cerebral insulin resistance elevates the activity of mTOR localized at the lysosomal membrane (115). Activation of the AMPK pathway has been shown to have potential benefits in protecting age-related neurodegenerative diseases, as AMPK can promote the lysosomal degradation of Aβ by reducing mTOR activity (116).

lysosomal acidification disorders was found to occur prior to Aβ deposition in AD mice, which is associated with a significant decrease in V-ATPase activity (117). Additionally, V-ATPase plays a crucial role in the generation and degradation of Aβ, and promoting the function of V-ATPase can significantly improve AD (118). Further studies have shown that in neurons exposed to AD pathogenic proteins and AD mice, the activity of V-ATPase in endolysosomes is reduced, specifically manifested by the binding of V-ATPase subunits ATP6V0C and ATP6V1B2 to Aβ and Tau toxic proteins *in vitro* and *in vivo*. Blocking the interaction between V-ATPase and Aβ or Tau proteins can be a specific approach to regulate V-ATPase and alleviate AD pathology (119). In the hyperglycemia state of DM, a reduction in the assembly efficiency of V-ATPase V0 and V1 subunits at neuronal synapses contributes to the occurrence of AD (120). The assembly of V-ATPase subunits affects its activity, thus suggesting that the decreased activity of V-ATPase plays a pivotal role in the occurrence and development of AD in patients with DM. Recent research further indicates that mutations in the CLEAR sequence of the V1 subunit of V-ATPase negatively affect the TFEB-V-ATPase axis, leading to Tau protein clearance disorders and accelerating neurodegeneration. This suggests that activating the upstream factor TFEB represents a potential novel therapeutic strategy for AD (121).

As a genetically predisposed disease, the genetic mechanisms underlying AD have also received extensive attention. A genetic analysis of 7,983 individuals revealed that carrying the CTSD allele rs17571 significantly increases the risk of AD (122). The hydrolase CTSD is involved in the degradation of Aβ and Tau proteins (123). Mice with CTSD gene knocked accumulate hyperphosphorylated tau protein at about three weeks of age, resulting in significant tau proteinopathy (124). APOE4 is an isoform of apolipoprotein E encoded by the ϵ4 alleles of the human APOE alleles, and is primarily involved in systemic lipid transport and the regulation of cholesterol homeostasis in the brain (125). In the early stages of AD, APOE4 may trigger the pathological cascade by reducing Aβ clearance, disrupting lysosomal homeostasis, and promoting lipid droplet accumulation in microglia to further accelerate Aβ deposition (126).In the context of T2DM, the carriage of ApoE4 exacerbates AD neuropathology. ApoE4 can induce cholesterol deposition in lysosomes, impair the lysosome dependent clearance of damaged mitochondria, and induce glucose metabolic disorders in astrocytes, significantly increasing the risk of AD (127). During AD progression, APOE4 acts synergistically with Aβ to exacerbate tau hyperphosphorylation and neuroinflammation, resulting in synaptic damage (128). In addition, APOE4 impairs the integrity of neuronal lysosomes in patients with AD, leading to the leakage of CTSD into the cytoplasm. This leaked CTSD degrades the cytoplasmic antioxidant protein thioredoxin 1, promoting neuronal apoptosis (129). In summary, CTSD and ApoE4 genes, as genetic background factors, contribute to AD by directly or indirectly affecting the function of neuronal lysosomes.

### 5.5 Diabetic retinopathy

Diabetic retinopathy (DR) is a serious complication of diabetes and one of the main causes of blindness in patients with DM. Currently, the pathophysiological mechanism of DR has not been fully elucidated (115). Previous studies have shown that abnormal autophagy levels accelerate the occurrence of DR (130), while lysosomal function closely affects the autophagy process. Therefore, the connection between DR and lysosomal dysfunction need to be further studied. The retina is composed of retinal pigment epithelial cells, glial cells (such as Müller cells), vascular cells (such as vascular endothelial cells), etc., which play different roles in maintaining the normal function of the retina.

The progressive dysfunction of retinal pigment epithelial cells plays a key role in the pathogenesis of DR (131). In the hyperglycemic state, the expression of CTSB in retinal pigment epithelial cells is upregulated, leading to LMP and the release of a large amount of CTSB, inducing dysfunction of the lysosomal-autophagy pathway and ultimately increasing apoptosis in retinal pigment epithelial cells (132). In the early stage of diabetes, the secretion of neurodegenerative factor glial maturation factor-β (GMFB) in the vitreous increases, leading to the translocation of ATP6V1A and preventing its assembly, disrupting lysosomal acidification and causing ferroptosis of retinal pigment epithelial cells. Further research has found that the death receptor FAS antagonist block GMFB induced lysosomal dysfunction in retinal pigment epithelial cells, providing a novel potential therapeutic target for early DR (133, 134).

Dysfunction of Müller cells in the human retina represent a key step in the development of diabetic macular edema (135). In studies on DM rats, it was found that the activity of CTSL in Müller cells decreased in the early stage of DR, leading to autophagy dysfunction, resulting in the generation of a large amount of vascular endothelial growth factor and Müller cell apoptosis. Rapamycin, an inhibitor of mammalian target of rapamycin (mTOR) and also an autophagy activator has the potential to improve DR by improving lysosomal function, reducing the production of vascular endothelial growth and Müller cell apoptosis (136, 137).

Hyperglycemia induces retinal vascular endothelial cells injury through multiple pathways. At present, improving endothelial cell function is the only successful clinical therapeutic target for DR (138).The decreased expression of CTSB and CTSD in retinal endothelial cells induced by hyperglycemia leads to autophagy disorders and pro apoptotic effects, thereby promoting vascular lesions in proliferative DR (139). Furthermore, the expression of TFEB, a major factor regulating lysosomal biogenesis in retinal vascular endothelial cells, is also reduced. Overexpression of TFEB may upregulate LAMP1 expression, promote lysosomal biogenesis, enhance autophagic flux, and protect these cells against oxidative stress and other injuries (140).

In summary, lysosomal dysfunction can affect different cells in the retina, leading to DR. This also suggests that improving lysosomal function during the progression of DR may have a better prognosis than a single therapeutic target, but this still needs to be confirmed by further research.

### 5.6 Diabetes related periodontitis

Diabetes-associated periodontitis (DP) is a chronic inflammatory periodontal tissue destructive disease associated with diabetes (141). Its clinical manifestations include destruction of periodontal supporting tissues, formation of periodontal pockets and loss of alveolar bone (142). DP is currently considered as “the sixth major complication of diabetes” (143). While diabetes increases the risk of periodontitis, periodontitis also negatively affects glycemic control (144). Autophagy is considered as a key step in defense against bacterial infection and excessive inflammatory response in periodontitis (145). Previous studies have shown that hyperglycemia aggravates periodontal inflammation by affecting autophagy, causing mitochondrial dysfunction and promoting macrophage pyroptosis (146). Given the critical role of lysosomal function in autophagy, the association between DP and lysosomal dysfunction deserves further investigation.

*Porphyromonas gingivalis* (*Pg*) is the main pathogen of periodontitis (147). *Pg* promotes the development of T2DM by inducing insulin resistance in adipose tissue, and glycated hemoglobin in the hyperglycemic environment further enhances the pathogenicity of *Pg (*148, 149). DM disrupts the dynamic balance between osteoblasts and osteoclasts, leading to pathological resorption of alveolar bone (150). Dihydroceramide phosphatidylglycerol produced by *Pg* induces LMP in osteoclast precursor cells, enhances the expression and activity of CTSB, and promotes the osteoclastogenesis (151). This suggests that *Pg* may contribute to periodontal bone destruction by regulating lysosomal function (151). In addition, *Pg* may induce PINK1-Parkin mediated mitophagy in oral epithelial cells, thereby triggering lysosome efflux and intracellular lysosome depletion while blocking autophagic maturation. This process may contribute to the extracellular release of lysosomal proteases, which could further exacerbate periodontal tissue destruction and alveolar bone loss (152). On the other hand, antimicrobial peptide LL-37 significantly upregulated the expression of lysosomal membrane protein LAMP-3 and promoted autophagy in keratinized epithelium to eliminate intracellular Pg (153). In summary, *Pg* promotes DP by negatively modulating the quantity and function of lysosomes, and enhancing autophagy by regulating lysosomal function, thereby eliminating *Pg* may serve as a novel therapeutic direction for periodontitis.

The gingival epithelial cells act as the first line of defense against bacterial infection, maintaining the health of the periodontal tissue (154). Hyperglycemia impairs the acidic environment of lysosomes by downregulating ATP6V0C expression, block the fusion between autophagosomes and lysosomes in gingival epithelial cells, and ultimately exacerbating periodontitis (155). The single-cell RNA sequencing of gingival tissues obtained from patients with T2DM has revealed that hyperglycemia induced lysosomal dysfunction by downregulating the expression levels of lysosomal LAMP2 and ATP6V0C, thereby disrupting autophagy and ultimately impairing gingival epithelial cell function (156). Hyperglycemia impairs the gingival epithelial cells while accelerating the apoptosis of periodontal supporting tissue cells such as periodontal ligament fibroblasts, leading to tooth loss (157). Against this pathological backdrop, periodontal ligament stem cells (PDLSCs) have emerged as a key research direction for periodontal tissue regeneration in periodontitis (158). The inflammatory environment in DM impairs the lysosomal degradative function of PDLSCs and inhibits the activity of TFEB, thereby reducing the osteogenic potential of PDLSCs (159, 160). Collectively, these findings indicate that hyperglycemia impairs the periodontal barrier and inhibit tissue regeneration capacity by disrupting the function of lysosomes, thereby exacerbating the pathological progression of periodontitis.

In summary, hyperglycemia and *Pg* jointly promote the pathogenesis and progression of DP by disrupting the function of lysosomes. Targeted modulation of lysosomal function may improve the therapeutic efficacy in patients with DP.

## 6 Conclusion and perspective

**Figure 3:**
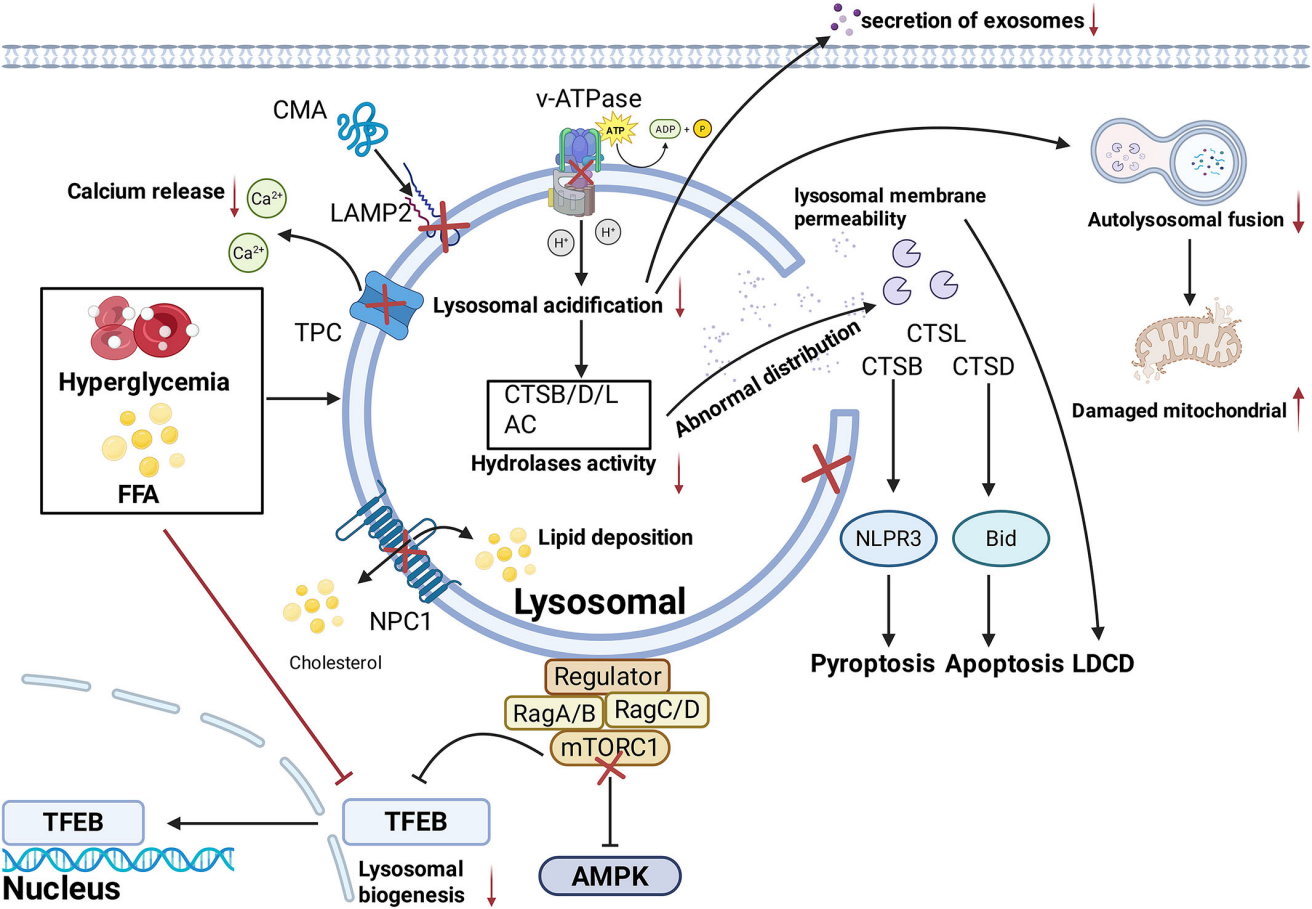
Relevant Pathways of Lysosomal Dysfunction in Diabetes.

Current research demonstrates that DM induced lysosomal functional alterations, are involved in the pathogenesis of diabetes and its chronic complications, including alterations in intraluminal pH (e.g., decreased activity of the V-ATPase), dysregulated expression of lysosomal hydrolases (e.g., downregulation or upregulation of CTSB, CTSL and CTSD), and impaired autophagosome-lysosome fusion (e.g., decreased expression of the LAMP-2). DM can also impair the lysosomes biogenesis by inducing the downregulation of TFEB expression. As a downstream signal molecule of TFEB, mTOR can not only regulate the biogenesis of lysosomes, but also directly regulate the function of lysosomes through the AMPK pathway. Collectively, these molecular and functional perturbations triggered by DM manifest as structural impairments of lysosomes, and **Figure 3** summarizes the impairment of key lysosomal structures under diabetic conditions. Therefore, the improvement of lysosomal function or biogenesis, and subsequent improvement of the autophagy may represent a promising therapeutic target for the management of DM and its chronic complications. However, the regulatory mechanisms by which lysosomes modulate DM and its complications independent of the autophagy pathway warrant further investigation. Notably, the abnormal distribution of lysosomal hydrolases such as CTSD induced by LMP is closely associated with lysosome dependent cell death (LDCD) (161). Current studies have revealed that LDCD is involved in various pathophysiological changes including inflammation, aging, and cardiovascular diseases (162). However, the association between LDCD and DM and its complications remains to be fully elucidated.

Some common hypoglycemic drugs can improve the clinical outcomes and prognosis of patients with DM by targeting the aforementioned lysosomal components. A typical example is metformin, which reduces hepatic lipid deposition by inhibiting v-ATPase and activating AMPK pathway in hepatocytes, and increases intestinal secretion of glucagon-like peptide-1 to lower blood glucose levels (163). Dapagliflozin, a sodium-glucose cotransporter 2 inhibitor, reduces endothelial cell injury by reducing CTSB expression (164). However, lysosomal components may exhibit distinct expression patterns in different cell types under DM conditions; for instance, the expression level of CTSB in retinal pigment epithelium and vascular endothelial cells is opposite. Thus, how to precisely modulate the expression of lysosomal components across diverse cell types under the intervention of drugs to achieve multi-organ benefits warrants further investigation. In addition, autophagy activators are regarded as having potential therapeutic effects on diabetes, However, excessive activation of autophagy in the context of lysosomal dysfunction may lead to further accumulation of autophagy intermediates, which can exacerbate cellular dysfunction, and accelerate the pathological progression of diabetes and its chronic complications. Current studies have shown that acidified nanoparticles can target lysosomes to restore the lysosomal acidic environment and autophagy, thereby improving impaired glucose tolerance and hepatic steatosis (165). Thus, there is still room for further exploration of lysosome-targeted antidiabetic agents in the future (166).
